# RocA Regulates Phosphatase Activity of Virulence Sensor CovS of Group A *Streptococcus* in Growth Phase- and pH-Dependent Manners

**DOI:** 10.1128/mSphere.00361-20

**Published:** 2020-05-20

**Authors:** Chuan Chiang-Ni, He-Jing Chiou, Huei-Chuan Tseng, Chih-Yun Hsu, Cheng-Hsun Chiu

**Affiliations:** a Department of Microbiology and Immunology, College of Medicine, Chang Gung University, Taoyuan, Taiwan; b Graduate Institute of Biomedical Sciences, College of Medicine, Chang Gung University, Taoyuan, Taiwan; c Molecular Infectious Disease Research Center, Chang Gung Memorial Hospital, Taoyuan, Taiwan; d Division of Pediatric Infectious Diseases, Department of Pediatrics, Chang Gung Memorial Hospital, Taoyuan, Taiwan; University of Iowa

**Keywords:** RocA, CovR-CovS, pH, group A *Streptococcus*

## Abstract

The control of the virulence response regulator and sensor (CovR-CovS) two-component regulatory system in group A *Streptococcus* (GAS) strains regulates more than 15% of gene expression and has critical roles in invasive GAS infection. The membrane-embedded CovS has kinase and phosphatase activities, and both are required for modulating the phosphorylation level of CovR. Regulator of Cov (RocA) is a positive regulator of *covR* and also been shown to be a pseudokinase that interacts with CovS to enhance the phosphorylation level of CovR; however, how RocA modulates the activity of CovS has not been determined conclusively. Although the phosphorylation level of CovR was decreased in the *rocA* mutant in the exponential phase, the present study shows that phosphorylated CovR in the *rocA* mutant increased to levels similar to those in the wild-type strain in the stationary phase of growth. In addition, acidic stress, which is generally present in the stationary phase, enhanced the phosphorylation level of CovR in the *rocA* mutant. The phosphorylation levels of CovR in the CovS phosphatase-inactivated mutant and its *rocA* mutant were similar under acidic stress and Mg^2+^ (the signal that inhibits CovS phosphatase activity) treatments, suggesting that the phosphatase activity, but not the kinase activity, of CovS is required for RocA to modulate CovR phosphorylation. The phosphorylation level of CovR is crucial for GAS strains to regulate virulence factor expression; therefore, the growth phase- and pH-dependent RocA activity would contribute significantly to GAS pathogenesis.

**IMPORTANCE** The emergence of invasive group A streptococcal infections has been reported worldwide. Clinical isolates that have spontaneous mutations or a truncated allele of the *rocA* gene (e.g., *emm*3-type isolates) are considered to be more virulent than isolates with the intact *rocA* gene (e.g., *emm*1-type isolates). RocA is a positive regulator of *covR* and has been shown to enhance the phosphorylation level of intracellular CovR regulator through the functional CovS protein. CovS is the membrane-embedded sensor and modulates the phosphorylation level of CovR by its kinase and phosphatase activities. The present study shows that the enhancement of CovR phosphorylation is mediated via the repression of CovS’s phosphatase activity by RocA. In addition, we found that RocA acts dominantly on modulating CovR phosphorylation under neutral pH conditions and in the exponential phase of growth. The phosphorylation level of CovR is crucial for group A *Streptococcus* species to regulate virulence factor expression and is highly related to bacterial invasiveness; therefore, growth phase- and pH-dependent RocA activity and the sequence polymorphisms of *rocA* gene would contribute significantly to bacterial phenotype variations and pathogenesis.

## Abstract (precis)

The emergence of invasive group A streptococcal infections has been reported worldwide. Clinical isolates that have spontaneous mutations or a truncated allele of the *rocA* gene (e.g., *emm*3-type isolates) are considered to be more virulent than isolates with the intact *rocA* gene (e.g., *emm*1-type isolates). RocA is a positive regulator of *covR* and has been shown to enhance the phosphorylation level of intracellular CovR regulator through the functional CovS protein. CovS is the membrane-embedded sensor and modulates the phosphorylation level of CovR by its kinase and phosphatase activities. The present study shows that the enhancement of CovR phosphorylation is mediated via the repression of CovS’s phosphatase activity by RocA. In addition, we found that RocA acts dominantly on modulating CovR phosphorylation under neutral pH conditions and in the exponential phase of growth. The phosphorylation level of CovR is crucial for group A *Streptococcus* species to regulate virulence factor expression and is highly related to bacterial invasiveness; therefore, growth phase- and pH-dependent RocA activity and the sequence polymorphisms of *rocA* gene would contribute significantly to bacterial phenotype variations and pathogenesis.

## INTRODUCTION

Group A *Streptococcus* (GAS) is a human pathogen that causes diseases that range from mild pharyngitis and tonsillitis to life-threatening necrotizing fasciitis and toxic shock syndrome. Epidemiological analyses have shown that specific *emm* types of GAS, such as *emm*1 and *emm*3, are associated with severe manifestations. Lynskey et al. (1) showed that *emm*3-type isolates have unique mutations in the *rocA* gene that contribute to increased production of the hyaluronic acid capsule. Miller et al. (2) further suggested that a null mutant allele of the *rocA* gene is a contributing factor to the association of *emm*3-type GAS isolates with severe infections. In addition to being identified in *emm*3-type isolates, the truncation of the RocA protein was found in *emm*18-type isolates (3).

RocA was identified as the positive regulator of *covR* transcription (4). CovR is the intracellular response regulatory protein that is composed of a set of the two-component regulatory system with the membrane-associated sensor CovS (5, 6). CovS has both kinase and phosphatase activities to modulate the phosphorylation level of CovR (7–9). The phosphorylation level of CovR was decreased in the *rocA* mutant compared to that in the wild-type strain (2, 10). Miller et al. (2) showed that RocA only enhances the phosphorylation level of CovR in the presence of a functional CovS. The direct interaction between RocA and CovS was demonstrated previously (10, 11). Lynskey et al. (11) showed that the N-terminal transmembrane domains of RocA are essential and sufficient for RocA to bind to CovS and to modulate the phosphorylation of CovR and CovR-controlled gene expression (11). Jain et al. (10) further indicated that RocA is a pseudokinase and that both kinase and phosphatase activities of CovS are required for RocA to modulate CovR phosphorylation. Nonetheless, Horstmann et al. (8) proposed that RocA could modulate CovR phosphorylation through impairing the phosphatase activity of CovS. Therefore, how RocA modulates the activity of CovS is not conclusively known.

Similarly to the *covS* mutant, the levels of phosphorylated CovR are decreased in the *rocA* mutant compared to those in the wild-type strains (2, 10). The expression of CovR-controlled genes such as the *hasABC* operon (encoding the hyaluronic acid capsule) and *slo* (encoding streptolysin O [SLO]) are upregulated in the *rocA* and *covS* mutants compared to that in the wild-type strain (12). Therefore, both *rocA* and *covS* mutants are more resistant to phagocytic killing than is the wild-type strain (2, 12–14). Nonetheless, the phenotypes of *covS* and *rocA* mutants are not identical. For example, the expression of cysteine protease SpeB was repressed in the *covS* mutant but was not downregulated in the *rocA* mutant in the stationary phase of growth or in subcutaneous infection of mice (10, 12). In addition, the *rocA* mutant has a better survival fitness in the respiratory tract infection model than the *covS* mutant (10).

The present study shows that, unlike the *covS* mutant, the level of phosphorylated CovR in the *rocA* mutant was increased in the stationary phase and under acidic culture conditions. Our results suggest that RocA enhances CovR phosphorylation through inhibiting the phosphatase activity of CovS in the exponential phase or under neutral pH culture conditions. The phosphorylation level of CovR is crucial for regulating virulence factor expression in GAS strains; therefore, the *rocA* mutant or the RocA-truncated isolates (e.g., *emm*3 and *emm*18 isolates) would produce higher levels of CovR-controlled virulence factors in the exponential phase or under neutral pH conditions than the RocA-intact isolates (e.g., *emm*1 isolates). This property would contribute significantly to GAS pathogenesis.

## RESULTS

### Roles of RocA in modulating CovR phosphorylation in different phases of growth.

**FIG 1:**
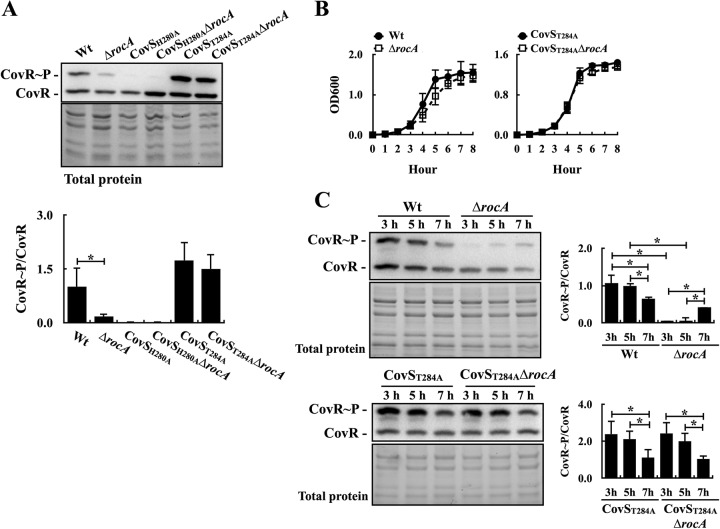
Phosphorylation levels of CovR in the wild-type strain (A20), the CovS kinase-inactivated mutant (CovS_H280A_), the CovS phosphatase-inactivated mutant (CovS_T284A_), and their *rocA* mutants (Δ*rocA*). (A) Phosphorylation levels of CovR in the A20, CovS_H280A_ mutant, CovS_T284A_ mutant, and their *rocA* mutants. Bacteria were cultured to the exponential phase of growth, and total proteins were extracted for Phostag Western blot analysis. (B) Growth curves of A20, the CovS_T284A_ mutant, and their *rocA* mutants. OD_600_, optical density at 600 nm. (C) Phosphorylation levels of CovR in A20, the CovS_T284A_ mutant, and their *rocA* mutants. Total proteins were extracted from bacteria cultured for 3 to 7 h and analyzed by Phostag Western blotting. CovR∼P, phosphorylated CovR; CovR, nonphosphorylated CovR. Total protein was used as the loading control. *, *P < *0.05.

CovS has both kinase and phosphatase activities and is required for RocA to modulate the phosphorylation level of CovR (7, 8). In line with results of a previous study (12), the Phostag Western blot analysis showed that the phosphorylation level of CovR was decreased in the *rocA* mutant compared to that in the wild-type A20 strain (Fig. 1A). Deletion of the *rocA* gene in the CovS kinase-inactivated (CovS_H280A_) mutant or the phosphatase-inactivated (CovS_T284A_) mutant (6, 8) did not influence the phosphorylation level of CovR in comparison with that of the parental strains (Fig. 1A), supporting the hypothesis that that RocA modulates CovR phosphorylation through CovS.

How RocA influences the phosphorylation level of CovR in the different growth phases was further elucidated. The kinase activity of CovS is essential for the optimal CovR phosphorylation; therefore, how RocA influences the phosphorylation level of CovR cannot be evaluated in the CovS_H280A_ mutant. The growth activity of the wild-type A20 strain, the CovS_T284A_ mutant, and their *rocA* mutants was similar (Fig. 1B). The phosphorylation level of CovR in the CovS_T284A_ mutant was gradually decreased from the lag phase (3 h) to the stationary phase (7 h) (Fig. 1C), suggesting that phosphatase activity of CovS was not involved in the decrease of CovR phosphorylation in the stationary phase. The phosphorylated CovR in the A20 *rocA* mutant was more abundant in the stationary phase than in the lag and exponential phases (Fig. 1C). In addition, the phosphorylation levels of CovR in A20 and its *rocA* mutant in the stationary phase of growth were comparable (Fig. 1C). These results suggest that RocA has a minor role in modulating CovR phosphorylation in the stationary phase of growth.

### Phosphorylation level of CovR in the wild-type strain and the *rocA* mutant in the different growth phases under Mg^2+^ treatments.

**FIG 2:**
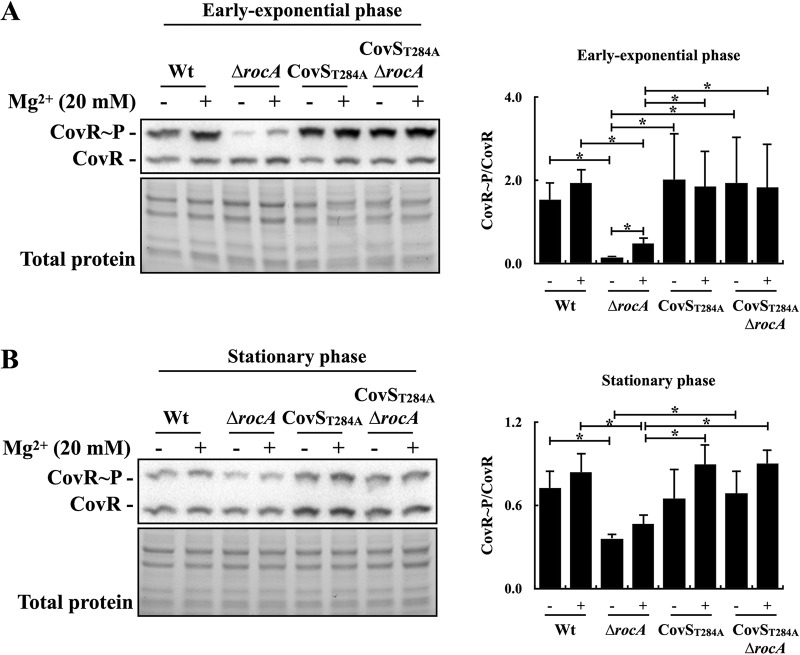
The phosphorylation level of CovR in the exponential-phase and stationary-phase wild-type strain (A20), the CovS phosphatase-inactivated mutant (CovS_T284A_), and their *rocA* mutants (Δ*rocA*) after Mg^2+^ stimulus. Bacteria were cultured to (A) the exponential phase (4 h of incubation) or (B) the stationary phase (6 h) and treated or not treated with 20 mM Mg^2+^ for an additional 1 h. After treatment, the total proteins were extracted and analyzed by Phostag Western blotting. CovR∼P, phosphorylated CovR; CovR, nonphosphorylated CovR. Total protein was used as the loading control. *, *P < *0.05.

Mg^2+^ is the signal to enhance CovR phosphorylation through inactivating the phosphatase activity of CovS (7). Therefore, the role of RocA in response to Mg^2+^ stimuli in the exponential and stationary phases was evaluated. Strain A20 and its *rocA* mutant were cultured to the exponential and stationary phases and treated with 20 mM Mg^2+^ for 1 h before the Phostag Western blot analysis. In the exponential phase of growth, the phosphorylation level of CovR was increased in A20 and its *rocA* mutant under Mg^2+^ treatments (Fig. 2A); however, the increase of phosphorylated CovR under Mg^2+^ stimuli was more pronounced in the *rocA* mutant compared to that in the wild-type A20 strain (Fig. 2A). In the stationary phase of growth, the phosphorylation level of CovR had no significant changes in the presence or absence of Mg^2+^ stimuli in both A20 and the *rocA* mutant (Fig. 2B). In addition, the Mg^2+^ treatment of the CovS_T284A_ mutant and its *rocA* mutant did not have significant effects on the phosphorylation level of CovR (Fig. 2A and B). These results suggest that the phosphatase activity of CovR is required for RocA to modulate the CovR phosphorylation and that phosphatase activity might be derepressed in the *rocA* mutant. In addition, the repression of CovS phosphatase activity by RocA was only observed in the exponential phase of growth.

### Phosphorylation level of CovR in the *rocA* mutant under neutral and acidic pH conditions.

**FIG 3:**
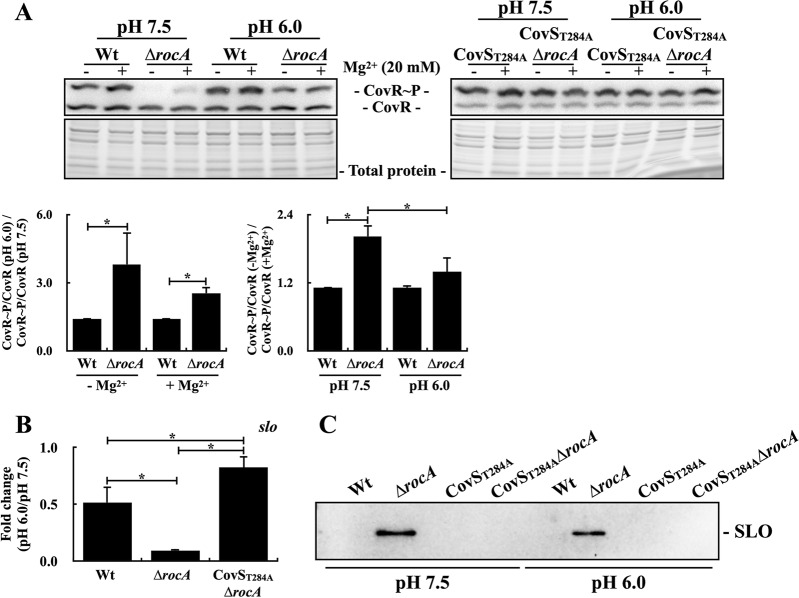
The phosphorylation level of CovR and the expression of CovR-controlled SLO in the wild-type strain (A20), CovS phosphatase-inactivated mutant (CovS_T284A_), and their *rocA* mutants (Δ*rocA*) under different pH and Mg^2+^-treatment conditions. (A) The phosphorylation level of CovR in A20, CovS_T284A_ mutant, and their *rocA* mutants after neutral and acidic broths treatments. Bacteria were cultured for 2 h in TSBY broth and treated with neutral and acidic broths (with or without 20 mM Mg^2+^) for an additional 1 h before the Phostag Western blot analysis. Total protein was used as the loading control. (B) Transcription and (C) expression of streptolysin O (SLO) in A20, its *rocA* mutant, and the CovS_T284A_ *rocA* mutant after neutral and acidic broth treatments. Bacteria were cultured for 2 h in TSBY broth and treated with neutral and acidic broth for an additional 1 h. RNA was extracted for reverse transcription-PCR (RT-qPCR) analysis, and bacterial culture supernatants were collected for detecting SLO by Western blotting. Biological replicate experiments were performed using three independent preparations. The expression of *slo* was normalized to that of *gyrA*. *, *P < *0.05.

Our previous study showed that acidic stress represses the transcription of CovR-controlled genes through CovR-CovS (15). Therefore, in addition to Mg^2+^, acidic stress could be the signal to inactivate the phosphatase activity of CovS. Results shown in Fig. 2 suggested that the phosphatase activity of CovS was derepressed in the *rocA* mutant in the exponential phase; therefore, whether CovS in the *rocA* mutant is more susceptible to the pH changes than that in the wild-type strain was further analyzed. The wild-type A20 strain and its *rocA* mutant were cultured to the early exponential phase and treated with neutral and acidic broth for an additional 1 h before the Phostag Western blot analysis. In the wild-type A20 strain, the phosphorylation level of CovR was slightly increased under acidic conditions compared to that under neutral pH conditions (Fig. 3A). The phosphorylated CovR cannot be detected in the *rocA* mutant under neutral pH conditions; however, the level of phosphorylated CovR under acidic conditions was increased about 3-fold compared to that under neutral pH conditions (Fig. 3A). Under neutral pH conditions, the phosphorylation level of CovR in the *rocA* mutant was increased upon Mg^2+^ stimuli (Fig. 3A). Nonetheless, under acidic pH conditions, the Mg^2+^ treatments had minor effects on enhancing the phosphorylation level of CovR in A20 and its *rocA* mutant (Fig. 3A). Notably, the phosphorylation level of CovR in response to Mg^2+^ stimuli in A20 and its *rocA* mutant under neutral and acidic culture condition were similar to those in the exponential and stationary phases, respectively (Fig. 2 and Fig. 3A). The phosphorylation levels of CovR in the CovS_T284A_ mutant and its *rocA* mutant were similar in the presence or absence of Mg^2+^ stimuli under both neutral and acidic conditions (Fig. 3A).

The changes in the phosphorylation level of CovR in strains with or without RocA under neutral and acidic culture conditions were further evaluated by detecting the CovR-controlled *slo* expression. In line with the results shown in Fig. 3A, the transcription of *slo* in the acidic culture condition showed a 10-fold and a 2-fold decrease in the *rocA* mutant and the wild-type A20 strain, respectively, in comparison with those in neutral pH conditions (Fig. 3B). In addition, the transcription of *slo* was similar in the CovS_T284A_ *rocA* mutant under neutral and acidic culture conditions (Fig. 3B). Finally, the expression of SLO was compared in the wild-type A20 strain, CovS_T284A_ mutant, and their *rocA* mutants under pH 7.5 and pH 6.0 conditions. SLO can only be detected in the culture supernatants of the *rocA* mutant, and the amount of SLO was decreased after acidic broth treatments compared to that treated by the broth with neutral pH (Fig. 3C). These results indicated that the phosphatase activity of CovS is more susceptible to pH changes in the *rocA* mutant than in the wild-type strain.

### Phosphorylation level of CovR and expression of CovR-controlled virulence factors in the *rocA trans*-complementary strain.

**FIG 4:**
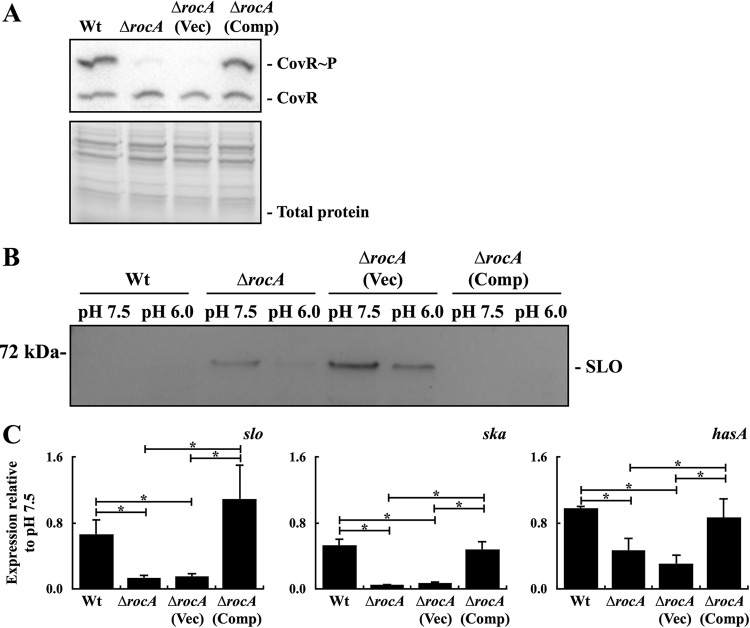
The phosphorylation level of CovR and the expression of CovR-controlled *slo*, *ska*, and *hasA* expression in the wild-type A20 strain, its *rocA* mutant (Δ*rocA*), and the vector control (Vec), and *rocA trans*-complementary strains (Comp). (A) The phosphorylation level of CovR in A20, its *rocA* mutant, and the vector control and *rocA trans*-complementary strains. Bacteria were cultured to the exponential phase of growth, and total proteins were extracted and analyzed by Phostag Western blotting. CovR∼P, phosphorylated CovR; CovR, nonphosphorylated CovR. Total protein was used as the loading control. (B) The expression of SLO and (C) the transcription of CovR-controlled *slo*, *ska*, and *hasA* in A20, its *rocA* mutant, and the vector-control and *rocA trans*-complementary strains after neutral and acidic broth treatments. Bacteria were cultured for 2 h in TSBY broth and treated with neutral and acidic broths for an additional 1 h. Bacterial culture supernatants were collected for detecting SLO by Western blotting, and RNAs were extracted for reverse transcription-quantitative PCR (RT-qPCR) analysis. Biological replicate experiments were performed using three independent preparations. The expression of *slo*, *ska*, and *hasA* was normalized to that of *gyrA*. *, *P < *0.05.

To verify that the phenotypes observed in the previous experiments were mediated by RocA, the *rocA trans*-complementary strain was constructed, and the phosphorylation level of CovR and the expression of CovR-controlled SLO in this complementary strain were analyzed. Results showed that the level of phosphorylated CovR was increased to that of the wild-type strain in the *rocA trans*-complementary strain but not in the *rocA* mutant and the vector-controlled strain (Fig. 4A). The SLO expression of these strains was first analyzed by Western blotting. In line with the results shown in Fig. 3C, SLO expression was only detected in the culture supernatants from the *rocA* mutant and its vector-controlled strain (Fig. 4B). In addition, the amount of SLO was decreased under acidic conditions (Fig. 4B).

Next, the transcription of *slo* and the CovR-controlled *ska* and *hasA* in these strains was compared under neutral and acidic culture conditions. The transcription of *slo* and *ska* in the wild-type A20 strain was repressed about 2-fold under acidic culture conditions compared to that under neutral pH conditions (Fig. 4C). In the *rocA* mutant and its vector-controlled strain, the transcription of *slo* and *ska* was repressed about 10- to 20-fold under acidic conditions (Fig. 4C). The transcription of *hasA* was not significantly repressed after 1 h of acidic broth treatments in the wild-type A20 strain; however, about 2-fold repression of *hasA* expression was observed in the *rocA* mutant and its vector-controlled strain (Fig. 4C). Finally, the *trans*-complementation of *rocA* in the *rocA* mutant restored the expression pattern of these genes to the wild-type strain’s level under neutral and acidic conditions (Fig. 4C).

### Phosphorylation level of CovR and expression of CovR-controlled genes in the mutant with the truncated *rocA* allele.

The *emm*3-type strains have been shown to have the truncated *rocA* allele that contributes to the low levels of phosphorylated CovR (1). The results shown in Fig. 3 suggested that the change of CovR phosphorylation level in the *rocA* mutant was more sensitive to different pH conditions than that in the wild-type strain. These results suggest that the different phenotypes between *emm*1-type and *emm*3-type strains could be contributed to by, at least partially, the presence or absence of the functional RocA protein. The *emm*3-type isolate was rarely identified in Taiwan. The *emm*3-type isolate of our collection showed an impaired growth activity, and the phosphorylation level of CovR could not be restored after complementation with the *rocA* gene from the *emm*1-type strain (data not shown), suggesting that this *emm*3-type isolate has unidentified mutations in multiple loci.

**FIG 5:**
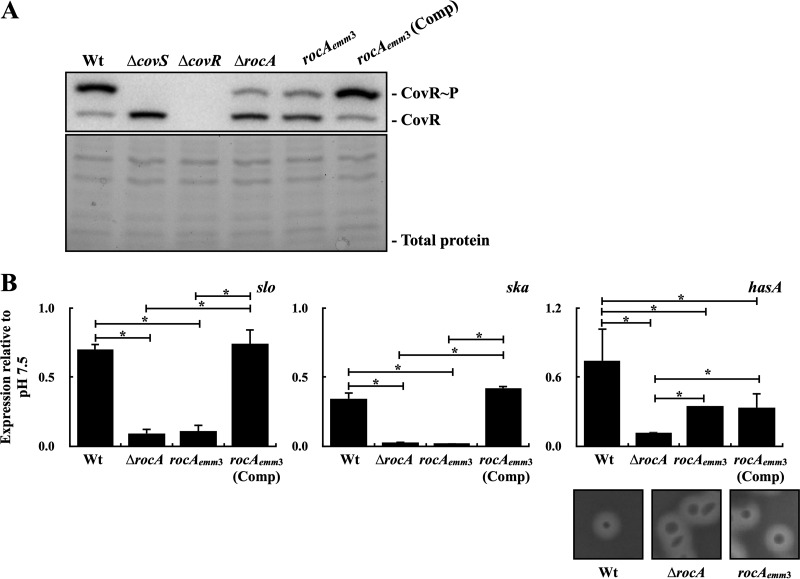
The phosphorylation level of CovR and the expression of CovR-controlled *slo*, *ska*, and *hasA* expression in the wild-type A20 strain, the *rocA*-deletion mutant (Δ*rocA*), the mutant with the truncated *rocA* allele from the *emm*3-type isolate (*rocA_emm_*_3_), and its *rocA trans*-complementary strain (Comp). (A) The phosphorylation level of CovR in A20, its *rocA* mutants, and the *rocA trans*-complementary strains. Bacteria were cultured to the exponential phase of growth, and total proteins were extracted and analyzed by Phostag Western blotting. CovR∼P, phosphorylated CovR; CovR, nonphosphorylated CovR. Total protein was used as the loading control. (B) The transcription of CovR-controlled *slo*, *ska*, and *hasA* in A20, its *rocA* mutants, and the *rocA trans*-complementary strain after neutral and acidic broth treatments. Bacteria were cultured for 2 h in TSBY broth and treated with neutral and acidic broths for an additional 1 h. Bacterial RNAs were extracted for RT-qPCR analysis. Biological replicate experiments were performed using three independent preparations. The expression of *slo*, *ska*, and *hasA* was normalized to that of *gyrA*. *, *P < *0.05. The lower panels show the colony morphology of A20 and its *rocA* mutants on the blood agar plate after 16 h of incubation at 37°C and under 5% CO_2_ conditions.

Instead, the *rocA* gene from the *emm*3-type isolate was amplified and utilized for replacing the *rocA* gene in the *emm*1-type A20 strain. The A20 strain with the truncated *rocA* gene (*rocA_emm_*_3_ mutant) showed lower levels of phosphorylated CovR compared to those of the parental A20 strain (Fig. 5A). In addition, *trans*-complementation with the *rocA* gene from A20 to its *rocA_emm3_* mutant restored the phosphorylation level of CovR (Fig. 5A). Next, the expression of CovR-controlled *slo*, *ska*, and *hasA* expression in the wild-type A20, its *rocA* mutant, the *rocA_emm_*_3_ mutant, and the *rocA trans*-complementary strain under neutral and acidic pH conditions were analyzed. Results showed that the transcription of *slo* and *ska* was more greatly repressed in the *rocA* deletion (Δ*rocA*) and *rocA_emm_*_3_ mutants compared to that in the wild-type A20 strain and the *rocA* complementary strain under acidic pH conditions (Fig. 5B). Nonetheless, unlike the transcription of *hasA* that was upregulated in the *rocA* mutant, the *rocA_emm_*_3_ mutant showed a similar level of the *hasA* transcription compared to that of the wild-type A20 strain (after 2 h of incubation and 1 h of neutral and acidic broth treatments) (data not shown), suggesting that the truncated RocA would retain partial regulatory activity in GAS strains. In line with these results, the *rocA* mutant showed a more mucoid colony morphology than that of the *rocA_emm_*_3_ mutant after 12 to 16 h of incubation (Fig. 5B, lower panels). Under the acidic condition, the *rocA* mutant showed greater repression of the *hasA* transcription than that of the *rocA_emm_*_3_ mutant. In addition, the *trans*-complementation of the *rocA* gene to the *rocA_emm_*_3_ mutant did not significantly influence *hasA* transcription under neutral and acidic pH conditions compared to that in its parental strain (Fig. 5B). These results suggest that the truncation of *rocA* in the *emm*3-type strain had a minor effect on the derepression of *hasA* expression compared to that in the *rocA* deletion mutant under this experimental condition.

## DISCUSSION

RocA is a pseudokinase and modulates the phosphorylation level of CovR through CovS (10, 16). The present study suggests that RocA enhances the phosphorylation level of CovR by inactivating the phosphatase activity of CovS. In addition, in the stationary phase or under acidic stress conditions, both RocA and the phosphatase activity of CovS have minor roles in modulating the phosphorylation level of CovR.

Although the direct interaction between RocA and CovS is essential for RocA to regulate CovR phosphorylation (11), how RocA modulates CovS activity is still not conclusively determined. Lynskey et al. (11) and Jain et al. (10) suggested that RocA enhances CovS kinase activity to result in increased CovR phosphorylation. Nonetheless, Horstmann et al. (8) proposed that RocA could form a complex with CovS and impair CovS phosphatase activity, resulting in increased CovR phosphorylation upon activation. The present study shows that the phosphorylation level of CovR and the expression of the CovR-controlled *slo* gene were similar in the CovS phosphatase-inactivated mutant (CovS_T284A_ mutant) and its *rocA* isogenic mutant under different culture conditions (Fig. 1A and C, Fig. 2, and Fig. 3). The CovS protein in the CovS_T284A_ mutant retains its kinase activity (Fig. 1A); therefore, these results supported the hypothesis that RocA would modulate CovR phosphorylation through inhibiting the phosphatase activity of CovS.

*In vitro* analysis showed that the phosphorylated CovR has a better DNA-binding activity to the *speB* promoter than that of the unphosphorylated CovR (17). Nonetheless, the transcription of *speB* is repressed by the nonphosphorylated CovR protein *in vivo* (18, 19). Although the phosphorylation level of CovR was decreased in both *covS* and *rocA* mutants compared to that in the wild-type strain, Feng et al. (12) showed that the *rocA* mutant, but not the *covS* mutant, expresses *speB* in the stationary phase of growth. Similar results were also reported by Jain et al. (10) in both M1 and M3 serotype strains. In line with these studies, we also detected the SpeB protein in the culture supernatant from the *rocA* mutant in the stationary phase (data not shown). In addition, in the stationary phase of growth, the phosphorylation levels of CovR in the wild-type strain, the CovS_T284A_ mutant, and their *rocA* mutants were comparable (Fig. 1C). These results suggest that RocA and the phosphatase activity of CovS have a limited role in modulating CovR phosphorylation in the stationary phase of growth. In addition, the level of nonphosphorylated CovR is crucial for repressing *speB* transcription (18); therefore, the increase of phosphorylated CovR in the stationary phase in the *rocA* mutant would trigger the transcription of *speB* to levels similar to those of the wild-type strain.

Unlike the *covS* mutants that are highly resistant to phagocytic killing, the CovS phosphatase-inactivated mutant, which has an increased level of phosphorylated CovR, cannot survive in human blood (8). These results suggest that GAS strains with a low level of phosphorylated CovR are more virulent and invasive. Nonetheless, the survival fitness of the *covS* mutants is decreased during pharyngeal infections compared to that of the wild-type strain (20–22). Although the phosphorylation level of CovR was decreased in both the *covS* and *rocA* mutants, Jain et al. (10) showed that the *rocA* mutant outcompeted the *covS* mutant in the competition assay of the *ex vivo* respiratory tract infection model. The importance of the cysteine protease SpeB in pharyngeal colonization was recently demonstrated (23). Therefore, these results suggest that the ability of the *rocA* mutant to phosphorylate CovR and secrete SpeB protease could be crucial for GAS to establish infections in the respiratory tract.

Horstmann et al. (7) showed that the M1 serotype (*emm*1-type) strains have higher levels of endogenous phosphorylated CovR than those in the M3 serotype (*emm*3-type) strains. In addition, treatments of the M3 serotype strain with Mg^2+^ (to inhibit the phosphatase activity of CovS) result in a greater increase of phosphorylated CovR than that in the M1 serotype strain (7). In the present study, we found that the increase of the phosphorylated CovR and the repression of CovR-controlled *hasA*, *ska*, and *slo* transcription in the *rocA* mutant under acidic conditions were more dominant compared with those in the wild-type strain under similar conditions (Fig. 4C). These results suggest that the difference between *emm*1 and *emm*3 strains would be caused, at least partially, by the truncation of the *rocA* allele in the *emm*3 strains. The truncated *rocA* alleles were found in the *emm*3 and *emm*18 isolates; in addition, the variations in the amino acid sequence of RocA among the *emm*28 isolates and in the number of tandem repeats in the promoter regions of *rocA* gene found in different *emm*89 isolates are associated with the changes of the regulatory activity of RocA and the expression level of *rocA*, respectively (24–26). These changes would result in the diverse responses when GAS invades into a niche with neutral pH conditions (e.g., blood) during infection and could be significantly associated with bacterial invasiveness.

**FIG 6:**
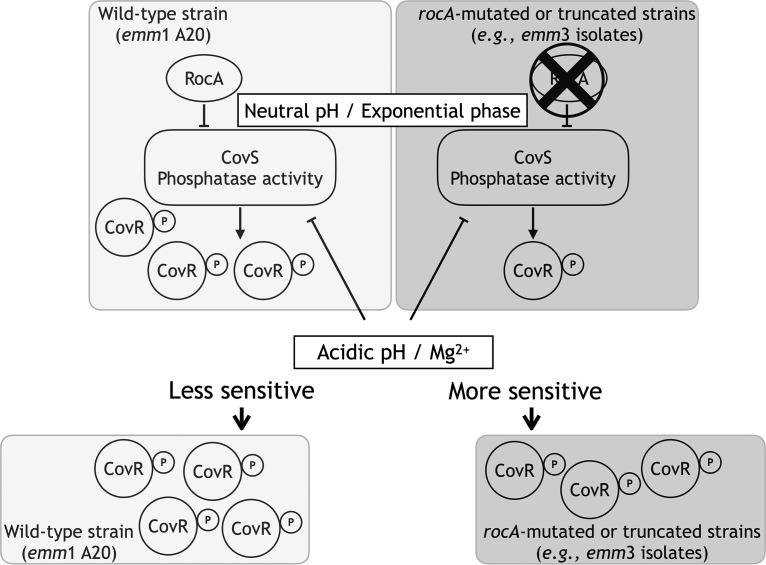
Model showing the difference in modulating CovR phosphorylation in the wild-type and *rocA*-mutated strains under Mg^2+^ or acidic pH stimuli. The diagram shows that RocA modulates the phosphorylation level of CovR via dominantly repressing CovS’s phosphatase activity under neutral pH and in the exponential phase of growth. In the *rocA*-mutated strains (the *rocA* isogenic mutant or RocA-truncated *emm*3-type isolates), the phosphatase activity of CovS is derepressed and resulted in low levels of phosphorylated CovR compared to those in the wild-type strain. Also, under acidic pH or Mg^2+^ stimuli (signals to inactivate the phosphatase activity of CovS), the increase of phosphorylated CovR is more pronounced in the *rocA*-mutated strains than that the wild-type strain, suggesting that RocA inactivation would result in increased susceptibility of GAS strains in response to changes of environmental conditions (e.g., pH, Mg^2+^, and growth phases).

In summary, results from the present study suggest that RocA modulates CovR phosphorylation via inhibiting the phosphatase activity of CovS under neutral pH conditions or in the exponential phase of growth. Clinical isolates that have spontaneous mutations or truncations in RocA would produce higher levels of SLO under neutral pH conditions and be more susceptible to environmental pH changes than strains with the intact *rocA* gene (Fig. 6); these properties could be significantly associated with the bacterial invasiveness and pathogenesis.

## MATERIALS AND METHODS

### Bacterial strains and culture conditions.

The GAS *emm*1-type A20 strain was described in a previous publication (27). The *emm*3-type isolate was provided by Jiunn-Jong Wu (School of Biomedical Science and Engineering, National Yang Ming University, Taiwan). GAS strains (Table 1) were cultured on Trypticase soy agar with 5% sheep blood or in tryptic soy broth (Becton, Dickinson and Company, Sparks, MD) supplemented with 0.5% yeast extract (TSBY). The neutral and acidic broths were prepared as described previously (15). For treating GAS strains with Mg^2+^ and neutral/acidic broths, bacteria were cultured to the early exponential phase (2 h after incubation) of growth. Bacterial pellets were collected by centrifugation (2,850 × *g* at 4°C), resuspended in fresh broth containing 20 mM Mg^2+^ or in neutral/acidic broths, and cultured at 37°C for another 1 h. Escherichia coli DH5α was purchased from Yeastern Biotech Co., Ltd. (Taipei, Taiwan) and was cultured in Luria-Bertani (LB) broth at 37°C with vigorous aeration. When appropriate, the antibiotics chloramphenicol (3 μg/ml and 25 μg/ml for GAS strains and E. coli, respectively) and spectinomycin (100 μg/ml) were used for selection.

**TABLE 1:** Plasmids and strains used in this study

Plasmid or strain	Description^*a*^	Reference or source
Plasmids		
pDL278	High-copy-number Escherichia coli-*Streptococcus* shuttle vector	31
pCN143	Temperature-sensitive vector	19
pCN172	pCN143::*rocA* Δ*cm*	This study
pCN181	pDL278::*rocA*	This study
pCN214	pCN143::*rocA_emm_*_3_	This study
Strains		
A20	*emm*1/ST28 wild-type strain	27
Δ*rocA*	A20 *rocA* isogenic mutant	This study
CovS_H280A_	A20 CovS_H280A_ mutant strain	15
CovS_H280A_ Δ*rocA*	The *rocA* isogenic mutant of the A20 CovS_H280A_ strain	This study
CovS_T284A_	A20 CovS_T284A_ mutant strain	9
CovS_T284A_ Δ*rocA*	The *rocA* isogenic mutant of A20 CovS_T284A_ strain	This study
*rocA_emm_*_3_	A20 with the truncated *rocA* allele from the *emm*3-type isolate	This study

a *cm*, chloramphenicol cassette; ST28, sequence type 28.

### DNA and RNA manipulations.

GAS genomic DNA extraction, RNA extraction, and reverse transcription were performed as described previously (9). Real-time PCR was performed in a 20-μl mixture containing 1 μl of cDNA, 0.8 μl of primers (10 μM), and 10 μl of SensiFast SYBR Lo-ROX premixture (Bioline Ltd., London, UK) according to the manual. Biological replicate experiments were performed from three independent RNA preparations in duplicate. The expression level of each target gene was normalized to *gyrA* and analyzed using the threshold cycle (ΔΔ*C_T_*) method (Applied Biosystems 7500 software v2.0.5; Thermo Fisher Scientific, Inc.). In addition, all values of control and experimental groups were divided by the mean of the control samples before statistical analysis (28). Primers used for real-time PCR analysis (Table 2) were designed with Primer3 v.0.4.0 (http://frodo.wi.mit.edu) according to the strain MGAS5005 sequence (NCBI reference sequence: CP000017.2).

**TABLE 2:** Primers used in this study

Primer	Use	Sequence (5′–3′)^*a*^	Reference or source
rocA-F-1	Construction	cg ggatcc tgttcccccatacagctagg	This study
rocA-R-1	Construction	cg ggatcc caacaccattgccatcactc	This study
rocA-F-3	Construction	tcc ccgcgg tgatgttaaaggtatgaata	This study
rocA-R-3	Construction	tcc ccgcgg catttatccttctccttctc	This study
rocA-F-4	Construction	gcg ggatcc ccgaggctgtcgtgaagtta	This study
rocA-R-4	Construction	gcg ggatcc tcagactcttaagttgattttgagtga	This study
rocA-F-5	Construction	cgc ggatcc aacgactgctcgttcaatca	This study
rocA-R-5	Construction	cgc ggatcc aaaatgccatcatgaatctgc	This study
slo-F-1	qPCR	gcagagcacaataaggtagt	15
slo-R-1	qPCR	ctggtgtatgaaataggataag	15
hasA-F-2	qPCR	tgaaagatctgacgctgacg	15
hasA-R-2	qPCR	accccaaaggcattatcgta	15
ska-F-1	qPCR	ttgctgacaaagatggttcg	19
ska-R-1	qPCR	ccctggtctgaaatcgtcat	19
gyrA-F-3	qPCR	cgtcgtttgactggtttgg	19
gyrA-R-3	qPCR	ggcgtgggttagcgtattta	19

a Underlining indicates restriction enzyme sites.

### Construction of the *rocA* mutants and the *trans*-complementary strain.

To construct the isogenic *rocA* mutant, the *rocA* gene with its upstream (723-bp) and downstream (569-bp) sequences were amplified by the primers rocA-F-1 and rocA-R-2 (Table 2). The PCR product (2,642 bp) was ligated into the temperature-sensitive vector pCN143 (19) with the BamHI site. The *rocA* gene in this plasmid was removed by PCR with the two reverse primers rocA-F-3 and rocA-R-3 and replaced by the chloramphenicol cassette from Vector-78 (29). The constructed plasmid, designated pCN172, was transformed into the wild-type A20 strain, and the transformants were selected as described previously (19). The deletion of the *rocA* gene in the selected transformants was confirmed by Sanger sequencing.

To introduce the *rocA* gene from the *emm*3-type isolate to the *emm*1-type A20 strain, the *rocA* gene of the *emm*3-type isolate was amplified by the primers rocA-F-5 and rocA-R-5 and ligated into the BamHI site of pCN143. The constructed plasmid, pCN214, was electroporated into the A20 strain as described previously (30) and cultured at 30°C with spectinomycin selection. The transformants were transferred to 37°C to force plasmid integration. Transformants in which plasmid was excised from the chromosome and replaced with the *rocA* gene from the *emm*3-type isolate were selected on the blood agar plate at 30°C. The sequence of the *rocA* gene of the transformant was further confirmed by Sanger sequencing.

To construct the *rocA trans*-complementary strain, the *rocA* gene with its native promoter (2,100 bp) was amplified from the *emm*1-type A20 strain by the primers rocA-F-4 and rocA-R-4 (Table 2). The PCR product was ligated into the Streptococcus-E. coli shuttle vector pDL278 (designated pCN181) and transformed into the *rocA* mutants. In addition, the empty pDL278 was electroplated into the *rocA* mutants as the empty vector-controlled strains.

### Western blot analysis for streptolysin O (SLO).

The overnight-cultured bacterial suspension was transferred (1:100) to fresh TSBY broth and cultured at 37°C for 2 h. The bacterial pellet was collected by centrifugation (2,850 × *g* at 4°C) and resuspended in Mg^2+^-containing or neutral/acidic broths for an additional 1 h at 37°C. Bacterial culture supernatants (30 μl) were mixed with 6× protein loading dye and separated by 12% SDS-PAGE. Separated proteins were transferred onto the polyvinylidene difluoride (PVDF) membrane (Millipore, Billerica, MA), and membranes were blocked by 5% skim milk in PBST buffer (PBS containing 0.2% Tween 20) at 37°C for 1 h. SLO was detected by the anti-SLO antibody (GeneTex, Irvine, CA). After hybridization, the membrane was washed with PBST buffer and hybridized with the secondary antibody, peroxidase-conjugated goat anti-rabbit IgG (1:10,000 dilution; Cell Signaling Technology, Inc., Danvers, MA) at room temperature for 1 h. The blot was developed using Pierce ECL Western blotting substrate (Thermo Fisher Scientific, Inc., Rockford, IL, USA). The signal was detected by the Gel Doc XR+ system (Bio-Rad), and the intensity of the detected bands was determined using Image Lab v6.0.1 (Bio-Rad).

### Phostag Western blot analysis.

Bacterial protein was extracted by a method described previously (19). Bacterial protein (10 μg) was mixed with 6× protein loading dye (without boiling) and separated by 10% SDS-PAGE containing 10 μM Phostag (Wako Pure Chemical Industries, Ltd., Richmond, VA) and 0.5 μM MnCl_2_. Phosphorylated and nonphosphorylated proteins were separated on Phostag SDS-PAGE gel for 120 to 140 min at 100 V at 4°C. Protein transfer, membrane blocking, hybridization, and signal detection were performed as described previously (19).

### Statistical analysis.

Statistical analysis was performed using Prism software v5 (GraphPad, San Diego, CA). Significant differences in multiple groups were determined using analysis of variance (ANOVA). Posttesting for ANOVA was done using Tukey’s honestly significant difference test. A *P* value of <0.05 was taken as significant.
